# Thoracoscopic Surgery for Glomus Tumor: An Uncommon Mediastinal Neoplasm and Iatrogenic Tracheal Rupture

**DOI:** 10.1155/2017/3621839

**Published:** 2017-01-04

**Authors:** Zhongjie Fang, Dehua Ma, Baofu Chen, Huarong Luo

**Affiliations:** ^1^Department of Cardiothoracic Surgery, Taizhou Hospital, Wenzhou Medical University, Linhai, Zhejiang, China; ^2^Department of Pathology, Taizhou Hospital, Wenzhou Medical University, Linhai, Zhejiang, China

## Abstract

Mediastinal glomus tumors are rarely recognized, and only seven cases have been reported in the literature. Here, we describe a rare mediastinal glomus tumor and review the characteristics of this rare clinical case. The patient was a 50-year-old female who presented with coughing for 3 months. Her chest computed tomography scan demonstrated a localized tumor in the posterior superior mediastinum. Intraoperatively, we found a longitudinal rupture of the membranous trachea above the carina. We completely resected the tumor and repaired the tracheal rupture under a thoracoscopy using a pedicled muscle flap. The tissue was diagnosed as a mediastinal glomus tumor according to its histological and immunophenotypic characteristics.

## 1. Introduction

Glomus tumor is a type of myoepithelial tumor that was first described by Hoyer in 1877. This type of tumor usually presents in locations that are rich in glomus bodies, including the subungual areas of the digits, arms, and feet, which are viewed as cutaneous structures that help to regulate body temperature and control blood pressure. In addition, glomus tumors have also been described in other locations, such as the trachea, gastric, esophagus, and pulmonary system [1]. However, glomus tumors located in the mediastinum are extremely rare, with only seven examples reported in the literatures. The simultaneous transmural rupture of the membranous trachea is rarely presented in these cases. Here, we describe a rare case of a posterior superior mediastinal glomus tumor. An iatrogenic rupture of the membranous trachea was found intraoperatively during tumor resection. The primary suture repair of the membranous tracheal wall defect was completed under a thoracoscopy.

## 2. Case Report

**Figure 1:**
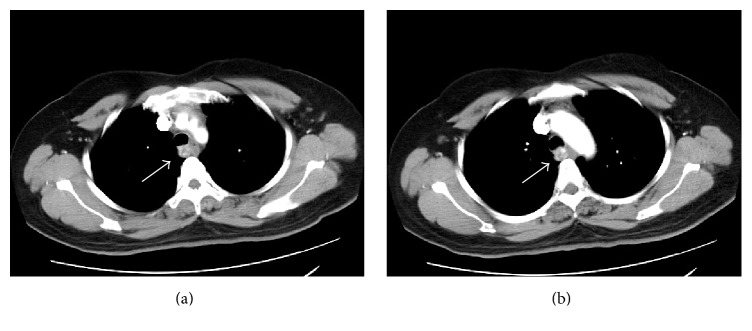
Thoracic computed tomography image showing an intensely enhanced lesion in the posterior superior mediastinum within the prevascular space.

A 50-year-old female repeatedly presented to our hospital with a cough for 3 months. No other symptoms were associated with the cough. The findings during the physical examination were unremarkable except that her Body Mass Index was 28.5. Contrast-enhanced computed tomography imaging of her chest revealed a posterior superior mediastinal lesion with an area of approximately 2.7 × 1.3 cm (Figure 1) and a value of approximately 122 HU. The mediastinal lesion manifested as an intensely enhancing mass with a circumscribed and well-defined margin. A bronchoscopy showed that the membranous trachea was raised, a mass oppressing the membranous region of the trachea from the posterior mediastinum. No pedunculated or polypoid tumor was in the trachea lumen.

**Figure 2:**
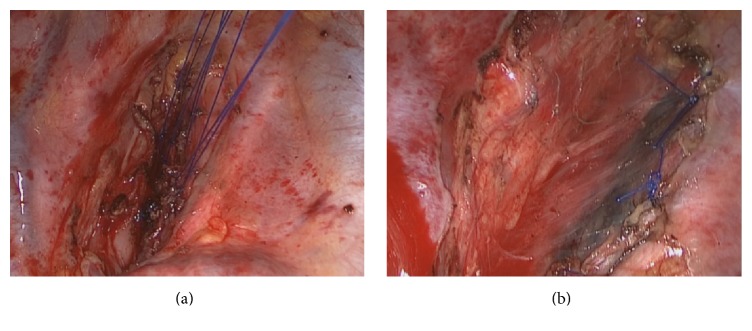
(a) Interrupted stitches using 4-0 Prolene. (b) Reinforcement with a pedicled pleural flap.

A surgical excision was performed using a right thoracoscopy. This procedure revealed a well-circumscribed vascular lesion that was located above the carina and behind the trachea that measured 1.5 cm in length and was highly vascularized. Additionally, the tumor closely adhered to the membranes of the trachea. During the process of tumor resection, to decrease tension, we emptied the balloon of an endotracheal tube with the assistance of anesthetist. After the lesion was resected, the endotracheal tube cuff was inflated and extruded into the chest. Regretfully, we ruptured the membranes in the trachea, which resulted in a longitudinal tear that was approximately 0.8 cm in length. This tear was sutured with interrupted stitches using 4-0 Prolene sutures and a pad of pleura. It was further reinforced with a pedicled pleural flap to avoid small amounts of air from leaking from the suture line (Figure 2). There was no air leakage when an airway inflation pressure of 20 cm of H_2_O was applied. The patient experienced an uneventful recovery and no postoperative complications and was discharged home on the 16th postoperative day. Fiberoptic bronchoscopy was performed 1 week, 3 months after surgery. No incidence of tracheostenosis occurred.

**Figure 3:**
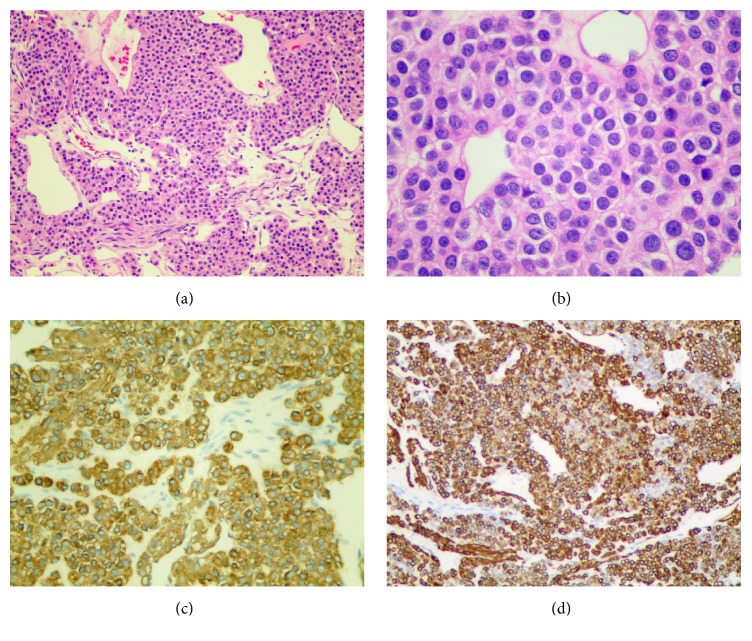
Histopathology showed that the tumor cells exhibited a sheet-like growth pattern with abundant eosinophilic cytoplasm and round nuclei. (a) Original magnification, 100x. (b) Original magnification, 400x. Immunohistochemistry for (c) SMA and (d) h-cald was positive.

On gross examination, the specimen was a relatively well-demarcated solid mass that measured 2.7 × 1.5 cm. The surface of a section of the tissue was reddish-grey and pliable in texture. Microscopically, the neoplastic cells contained a moderate amount of cytoplasm and small and round-oval nuclei, and a few mitotic structures unsystematically surrounded the vessel. Immunohistochemistry demonstrated that the cells were positive for SMA (smooth muscle actin) and h-cald and negative for CD34, Desmin, HMB45, CK, Syn, and S100 (Figure 3). The tumor was diagnosed as a mediastinal glomus tumor according to its histological and immunophenotypic characteristics.

## 3. Discussion

Glomus tumors are neuromyovascular, arteriovenous structures that can be located in the limb extremities in glomus bodies, which are thought to be involved in regulating blood flow and temperature. This type of tumor can also be located in other organs, including the pharynx, trachea, or stomach [1]. However, these tumors are rarely found in the mediastinum. A survey of the literature revealed that only seven cases have been previously described in the English literature [2–8]. According to the previous literature, the major symptom that is associated with this disease is chest pain or dyspnea. These symptoms were not observed in our patient, who was admitted with a cough that was potentially due to bronchial stenosis. Microscopically, these tumors contain branching vascular channels that are lined with endotheliocytes and interspersed by uniformly round or ovoid glomus cells. The lesion cells are lightly eosinophilic. Glomus tumors typically comprise 3 components: glomus cells, vasculature, and smooth muscle cells. These tumors can be subdivided according to their main component into three major classes. The most common (approximately 75%) is solid glomus tumors, which have poor vasculature and a scant smooth muscle component. The second most common type is glomangiomas which display a prominent vascular component. Finally, glomangiomyoma is the least common type. These structures have prominent vascular and smooth muscle components. Glomus tumors may be associated with an autosomal dominant mutation in a gene located on chromosome 1p [9]. Immunohistochemically, glomus cells are positive for smooth muscle actin, vimentin, and type IV collagen, and some glomus tumors may also show immunopositivity for desmin, caldesmon, and calponin.

Our case presents several significant characteristics that have not been previously observed. First, it is unique because the tumor was located in the upper posterior mediastinum, where it closely adhered to the membranous region of the trachea and esophagus above the carina. This is the most likely cause of her cough. These tumors share many imaging features with common mass lesions of the mediastinum, including thymic carcinoid tumors, pheochromocytomas, and hypervascular lymphadenopathies. They are therefore easily misdiagnosed. Accordingly, a mediastinal glomus tumor should be regarded as part of a differential diagnosis during an evaluation of mediastinal neoplasms. Finally, we identified the iatrogenic tracheal rupture intraoperatively. The causes of the tracheal rupture may have been directed trauma during the resection of the glomus tumor which may have been caused by its close adhesion to the membranous region of the trachea. It is worth mentioning that a postintubation tracheal rupture is a complication that is also observed during endotracheal intubation. The risk factors for this condition are female gender, obesity, age > 50 years old, tube cuff overinflation, and a sudden increase in tracheal pressure [10], almost all of which were observed in our patient. In a review [11] of longitudinal membranous tracheal wall injuries, especially those diagnosed intraoperatively, the top choice was to perform primary suture repair of the membranous tracheal wall defects and cover the suture line with tissue, such as pericardium, muscle, pleura, or the mediastinal fat pad, to reinforce the repair. It has been demonstrated that this method of repair is safe and effective when performed under a thoracoscope. In conclusion, thoracic surgeons and anesthesiologists must be alerted to the possibility for the tracheal rupture in patients intubated with double-lumen tube.

In summary, we described an extremely rare case of a mediastinal glomus tumor, which should be regarded as part of a differential diagnosis during an evaluation of mediastinal neoplasms. We successfully repaired the tracheal rupture under a thoracoscope using a pedicled muscle flap.
